# The family of LSU-like proteins

**DOI:** 10.3389/fpls.2014.00774

**Published:** 2015-01-13

**Authors:** Agnieszka Sirko, Anna Wawrzyńska, Milagros Collados Rodríguez, Pawel Sęktas

**Affiliations:** Institute of Biochemistry and Biophysics, Polish Academy of SciencesWarsaw, Poland

**Keywords:** *Arabidopsis*, tobacco, coiled coil, SALK mutants, gene expression, OAS, ethylene, UP9

## Abstract

The plant response to sulfur deficiency includes extensive metabolic changes which can be monitored at various levels (transcriptome, proteome, metabolome) even before the first visible symptoms of sulfur starvation appear. Four members of the plant-specific *LSU* (response to Low SUlfur) gene family occur in *Arabidopsis thaliana* (*LSU1-4*). Variable numbers of *LSU* genes occur in other plant species but they were studied only in *Arabidopsis* and tobacco. Three out of four of the *Arabidopsis LSU* genes are induced by sulfur deficiency. The *LSU*-like genes in tobacco were characterized as *UP9* (UPregulated by sulfur deficit 9). LSU*-*like proteins do not have characteristic domains that provide clues to their function. Despite having only moderate primary sequence conservation they share several common features including small size, a coiled–coil secondary structure and short conserved motifs in specific positions. Although the precise function of LSU-like proteins is still unknown there is some evidence that members of the LSU family are involved in plant responses to environmental challenges, such as sulfur deficiency, and possibly in plant immune responses. Various bioinformatic approaches have identified LSU*-*like proteins as important hubs for integration of signals from environmental stimuli. In this paper we review a variety of published data on *LSU* gene expression, the properties of *lsu* mutants and features of LSU-like proteins in the hope of shedding some light on their possible role in plant metabolism.

## INTRODUCTION

The first global analyzes of gene expression profiles under sulfur deficiency stress in *Arabidopsis* appeared in Hirai et al. (2003); Maruyama-Nakashita et al. (2003) and Nikiforova et al. (2003), however, these studies focused on genes encoding proteins with known functions. Two years later *LSU1* (At3g49580) and *LSU2* (At5g24660) were identified as two out of 15 sulfur-responsive genes which were significantly up-regulated in roots as early as 2 h (*LSU1*) or 4 h (*LSU2*) after plants were transferred to sulfur-free medium; a sulfur-responsive element (SURE) was identified in their promoter regions (Maruyama-Nakashita et al., 2005). In the same year the At3g49580 gene appeared on the list of important network elements identified in a pioneering study involving reconstruction of the gene-metabolite network involved in the plant response to sulfur deficiency stress (Nikiforova et al., 2005). At the same time the tobacco *UP9* gene was independently shown to be strongly and specifically up-regulated by sulfur deficiency (-S) using an unbiased suppression subtractive hybridization approach (Wawrzyńska et al., 2005). Since then rather few studies focusing on *LSU*-like genes and proteins have been published; however, several reports presented results of high throughput experiments which included also data related to the regulation of expression and phenotypes of the *Arabidopsis lsu* mutants. The systematic review of available data presented below provides clear evidence of the importance of this family of proteins and, hopefully contributes to uncovering their function.

## *LSU/UP9* GENES AND THEIR EXPRESSION

### *LSU* GENES IN *Arabidopsis*

**FIGURE 1:**
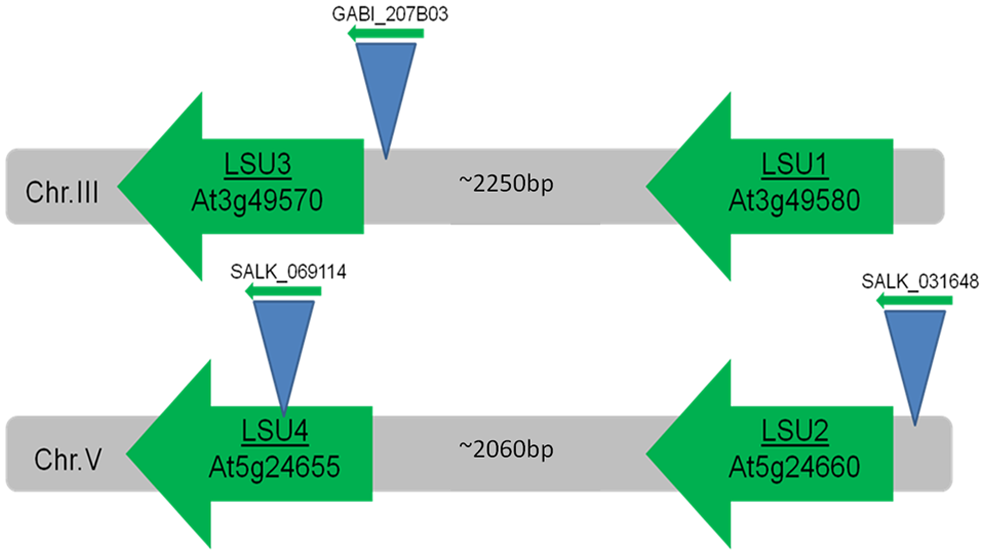
**Localization of ‘response to Low Sulfur’ (LSU) genes in the *Arabidopsis* genome.** Number of base pairs (bp) between LSU open reading frames is indicated. Positions of T-DNA inserts are shown schematically.

*Arabidopsis thaliana* contains four *LSU* genes (*LSU1*–*LSU4*) which are localized in pairs of direct repeats on two chromosomes (**Figure 1**). The nucleotide sequences of chromosome III corresponding to *LSU1* and *LSU3* transcripts are separated by about 2250 bp; the distance between *LSU2* and *LSU4* is slightly shorter (about 2060 bp). The open reading frames (ORFs) are relatively small and consist of about 280 bp. Most *LSU* genes have no introns; however, a spliced variant of *LSU1* (At3g49580.2) encoding a protein with internal deletion of 19 amino acids was reported [http://www.arabidopsis.org]. Searches of publicly available microarrays using the Genvestigator platform (Zimmermann et al., 2004, 2008) showed that *LSU1* and *LSU2* are strongly expressed under -S but *LSU4* appears not to be induced by -S. *LSU3* was not included in these microarrays. Expression of *LSU1* and *LSU2* is induced not only by -S but also by other stressful environmental conditions such as salt stress and AgNO_3_ treatment.

Several global analyzes of *Arabidopsis* gene expression in various growth conditions and developmental stages provide valuable information about expression of *LSU*s. Most of these data relate to *LSU2*, suggesting that this member of the family is preferentially involved in the plant response to certain stresses or certain processes. Expression of *LSU2* is induced during oxidative stress (Davletova et al., 2005) and at the beginning of an extended night, which may indicate that it is induced by carbon starvation and in response to sugar (Usadel et al., 2008). Recently it has been shown that expression of *LSU2* is induced by a combination of light and plastid signaling (Ruckle et al., 2012); these authors identified *LSU2* as one of seven so-called *END* (enhanced de-etiolation) genes. They went on to characterize some of the *end* mutants, including *lsu2* (SALK_031648), and showed that expression of some photosynthesis-related genes (*Lhcb1.4*, *PsbS*, *RbcS1,* and *CHS*) was attenuated in them. The mechanisms responsible for the regulation of transcription in *end* mutants remain unclear; the expression of *END* genes is regulated by a variety of signals besides light and plastid signals, so it may be different for different mutants. Ruckle et al. (2012) concluded that the products of *END* genes contribute to a complex network responsible for optimization of chloroplast function during chloroplast biogenesis, and probably during periods of chloroplast dysfunction. The link between *LSU2* and chloroplasts was also emphasized in a recent report, where *LSU2* was identified as one of 39 genes that were differentially expressed in six independent microarray experiments using plants with the provoked retrograde signaling in response to disturbances of chloroplast performance by chemical treatment or mutation of some metabolic pathways (Glasser et al., 2014).

In addition *LSU2* was tentatively identified as one of the genes involved in the crosstalk between several signals (nitrate, sulfur, iron, and hormones) from analysis of transcriptome data for *Arabidopsis* plants grown under sulfur and iron restriction, and various nitrate and stress hormone treatments (Omranian et al., 2012).

Somewhat surprisingly expression of *LSU1* was found to be repressed during infection with cabbage leaf curl virus (CaLCuV), whereas *LSU2* expression was apparently unaffected (Ascencio-Ibanez et al., 2008). The *LSU1* gene was also shown to be constitutively (phase-independently) expressed during pollen germination and tube growth (Wang et al., 2008).

Analysis of publicly available data from two sets of high-throughput experiments led to the identification of *LSU1* as a member of a six-gene cluster responding to O-acetylserine (OAS) levels in shoots (Hubberten et al., 2012b). One set of data was from experiments on diurnal oscillations of genes and metabolites (Espinoza et al., 2010); the second set was from studies of plants during the light-dark transition (Caldana et al., 2011). OAS was one of the compounds most affected by changes in conditions in both studies. Hubberten et al. (2012b) confirmed that regardless of temperature (20 or 4°C), the level of OAS (and the expression of the above-mentioned genes) increased during the night and decreased during the day. Increased expression of *LSU1* (and the other five genes) was also observed following induction of the chemically inducible ectopic copy of *SERAT* (encodes serine acetyltransferase, which is involved in OAS synthesis) in sulfur-sufficient transgenic plants (Hubberten et al., 2012b). The same group used a split-root approach to explore further the role of OAS in the regulation of plant S-status in *Arabidopsis.* One half of the root was exposed to -S, whilst the other half of the root of the same plant was grown in sulfur-sufficient conditions. OAS levels were low in both halves of the split root, and expression of previously mentioned OAS-responsive genes, including *LSU1* was also low (Hubberten et al., 2012a).

It has recently been reported that expression of *LSU1* (and *BGLU28* [At2g44460], *SDI1* [At5g48850] and *SULTR4;2* [At3g12520]) is much less affected by S availability in the *sultr1;2* mutants than in the wild type (Zhang et al., 2014). This observation is not strictly related to the function of LSU/UP9 proteins, nevertheless it is worth noting because it makes an important contribution to understanding the plant mechanisms responsible for sensing S availability and thus also S status-dependent regulation of gene expression.

**FIGURE 2:**
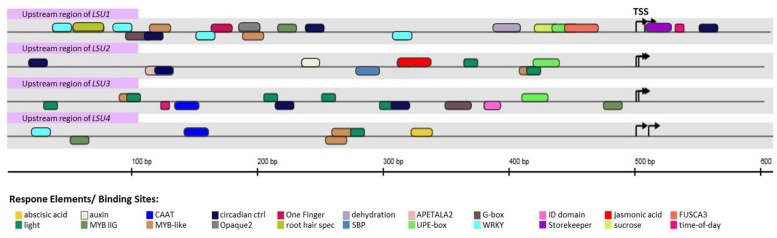
**Depiction of transcription factor (TF) binding sites found in the upstream regions of *LSU1-4*.** The sequences were scanned for matches to TFs binding sites using MatInspector, part of the Genomatix Software Suite (www.genomatix.de). A match is represented by a round-ended rectangle. Matches with the positive and negative strands are depicted above or below the sequence line, respectively. The arrow symbol on the sequence stands for a transcription start site (TSS); note that there are several putative TSSs for each *LSU*.

The results of *in silico* analysis of the promoter regions of the *LSU*s are shown in **Figure 2**. Analyzes of the 500 bp upstream transcription start site (TSS) demonstrate the potential for differential expression of each *LSU* gene. In all but the *LSU4* promoter, there is an element specific for induction in -S, UPE-box (Wawrzyńska et al., 2010). Additional sulfur-responsive elements (SURE boxes) which are not included in **Figure 2** have previously been identified in the promoter regions of *LSU1* and *LSU2* (Maruyama-Nakashita et al., 2005). The *LSU1* promoter contains the largest number of potential regulatory elements. Only *LSU1* has consensuses for FUSCA3 and OPAQUE2-like factors, both of which are essential for seed-specific expression (Moreno-Risueno et al., 2008) and the *cis*-elements related to response to dehydration and sucrose. The binding site for the bZIP transcription factors (TFs; G-box) is present in the *LSU1* and *LSU3* promoters, whilst the consensus for binding the WRKY TFs is present in *LSU1* and *LSU4*. The promoter regions of *LSU2, LSU3,* and *LSU4* (but not *LSU1*) have sequences for binding the light-responsive factors. The *LSU2* promoter contains sequences responsive to auxins and jasmonic acid as well as sequences for APETALA2 and SQUAMOSA promoter-binding protein (SBP), indicating that *LSU2* may play an important role in ontogenesis. The *LSU3* promoter contains a specific sequence which binds the INDETERMINATE1 (ID domain) responsible for the transition to flowering; the *LSU4* promoter has a *cis*-acting sequence responsive to abscisic acid (ABA). The putative roles of these elements in the regulation of *LSU* gene expression should be verified experimentally.

### *UP9* GENES IN TOBACCO

The tobacco LSU-like proteins were grouped into six clusters (UP9A to UP9F); however, the exact number of such genes in tobacco remains unclear (Lewandowska et al., 2010). Only one of these genes, *UP9C*, has been investigated further. An increase in the level of the *UP9C* transcript was observed just 2 days after transferring plants from sulfur-sufficient to sulfur-deficient medium in all parts tested (roots, young leaves, mature leaves, stems) and a further increase in transcript level was observed after additional days under -S. Analysis of the promoter region of *UP9C* indicated that it has only one TSS located 109 bp upstream of the translational start site (Wawrzyńska et al., 2010). The same study also reported the presence of an interesting motif, UPE-box, in the *UP9C* promoter. The authors used the DNA fragment containing UPE-box (from the promoter region of *UP9C*) in a yeast-one-hybrid experiment and identified NtEIL2, a tobacco member of the EIL family, as a TF which bound to the UPE-box (Wawrzyńska et al., 2010). Transient expression assays in *Nicotiana benthamiana* plants indicated that NtEIL2 was responsible for the UPE-box-dependent up-regulation of the reporter gene in -S conditions. Interestingly, an *Arabidopsis* homolog of NtEIL2, SLIM1, which has been identified earlier as a critical transcriptional regulator of plant sulfur response and sulfur metabolism (Maruyama-Nakashita et al., 2006), was also able to bind to *UP9C* promoter containing UPE-box. Mutations in UPE-box affect the binding of both factors, NtEIL2 and AtSLIM1; however, in the presence of SLIM1 the promoter was constitutively active, regardless of the plants’ sulfur status (Wawrzyńska et al., 2010). In conclusion, *UP9C* seems to be regulated directly by NtEIL2, in a sulfur-dependent manner. Some as yet unidentified species-specific factors guarantee the specificity of the NtEIL2-dependent up-regulation of the *UP9C* gene (and possibly other genes containing UPE-box) in -S conditions. Further *in silico* analysis of the promoter region showed that the *UP9C* promoter has elements which are potentially responsive to light, salt stress and phytohormones such as ABA, ethylene and cytokines as well as the above-mentioned SURE located 350 bp upstream of the start codon. The biological significance of these cis-factors is unknown.

UPE-box is also present in the promoters of several *Arabidopsis* genes (Wawrzyńska et al., 2010). A search of the genome sequence revealed that it was present in the promoter regions of *LSU1* (At3g49580), *LSU2* (At5g24660), and *LSU3* (At3g49570; but not *LSU4*) and also in several other genes which are up-regulated in -S. Interestingly the set of genes containing UPE-box in promoter appears to be very similar to the OAS cluster genes (Hubberten et al., 2012b).

## PHENOTYPES OF THE MUTANTS

### ANALYSIS OF *Arabidopsis* SALK MUTANTS

One of the difficulties in determining the function of proteins from the LSU family is that information about the phenotypes of knock-out (KO) and knock-down (KD) mutants is scarce. There are T-DNA insertional mutants for *LSU2* (e.g., SALK_31648, SALK_070105), *LSU3* (e.g., GABI_207B03), and *LSU4* (e.g., SALK_069114) but not for *LSU1* (**Figure 1**). The high probability of functional overlap makes it desirable to test multiple *lsu* KO or KD mutants, but so far no data have been published. Most available data relate to *lsu2* mutants, for example an interesting report on the functional characterization of abiotic stress response proteins with unknown function was published recently (Luhua et al., 2013). These authors tested the response to treatments such as salinity, oxidative, osmotic, heat, cold, and hypoxia stress of 1007 T-DNA insertional mutants in genes with unknown function. The *lsu2* mutant (SALK_31648C) was one of 69 genes with an unknown function that seemed to be more tolerant of osmotic stress than the wild type; responses to other stresses did not appear to be altered. Another study reported that *lsu2* mutants (SALK_031648, SALK_070105) exhibited enhanced susceptibility to two evolutionarily distinct pathogens, *Pseudomonas syringae* and *Hyaloperonospora arabidopsidis* (Mukhtar et al., 2011). According to the authors, LSU2 (and other proteins, for example JAZ3) has some effect on the functioning of the NB-LRR (nucleotide binding leucine-rich repeat) intracellular immune receptors with particular emphasis on the RPS2 (Resistance to *Pseudomonas syringae* 2) protein. Activation of NB-LRR proteins is responsible for robust disease-resistance responses such as host cell death and systemic defense signaling.

Defects in flower and inflorescence development were observed in the insertion mutant *lsu4* (SALK_069114) when grown under short-day conditions (Myakushina et al., 2009). Mutation of the *LSU4* gene caused delayed flowering and disturbances in the formation of flower organs. There were also significant changes in the expression of many regulatory genes, including down-regulation of *LEAFY* (*LFY*), *APETALA1* (*AP1*), *APETALA3* (*AP3*), *PISTILLATA* (*PI*) and *SEPALLATA3* (*SEP3*) and up-regulation of *APETALA2* (*AP2*), *AGAMOUS* (*AG*) and *SEPALLATA* (*SEP2*). It is worth mentioning that the authors noted that *LSU4* expression increased two to threefold under phosphorus, nitrogen, potassium, or iron deficiency.

### SILENCING OF *UP9* IN TOBACCO

Analysis of the tobacco antisense *UP9C* transformants (KD) revealed no evidence of phenotypic differences from the wild type, although the KD transformants did have a different metabolite profile from wild type plants (Lewandowska et al., 2010). The metabolite profiles of KDs grown in -S were more similar to the profiles of parental line plants grown in sulfur-sufficient conditions, suggesting that the KD lines failed to adjust their metabolism to the -S conditions. In addition the level of non-protein thiols (consisting mostly of glutathione) in mature leaves and roots, but not in young leaves, was different in KD plants. Wild type plants showed the expected reduction in glutathione levels in mature leaves 2 days after transfer to -S, but there was no change in the KDs, which had a high level of glutathione in the mature leaves regardless of the conditions. The mutants did, however, have low levels of glutathione in the roots, particularly under -S; mutants also had lower levels of sulfur in the roots under -S than the wild type. Another interesting observation was that under -S several genes were misregulated in the mutants; usually the level of transcription was lower in the KDs than the wild type. It must be remembered, however, that only a limited selection of genes was tested and no high-throughput analysis was performed in this study.

Ethylene plays a very important role in plant response to several stresses and regulates many processes (Adie et al., 2007; Lin et al., 2009). In -S conditions ethylene levels increase in wild type tobacco. KD plants have lower levels of ethylene than wild type plants in -S conditions (Moniuszko et al., 2013), but in sulfur-sufficient conditions the *UP9C*-silenced line produced slightly more ethylene than the wild type. Transcriptome analysis revealed significant changes in the gene expression pattern of the KD line relative to the wild type; only 130 of the 360 genes up-regulated in the wild type in -S were also up-regulated in the mutants and only 14 of 91 genes down-regulated in the wild type were also down-regulated in the mutant. Some genes were regulated in the mutant but not in the wild type. Differences in the expression profiles of the mutants and wild type may provide clues to function. Gene ontology (GO) analysis indicated clearly that UP9C does not participate in sulfur deficiency-dependent regulation of genes encoding isoforms of APS reductase (APR) or genes encoding S-adenosylmethionine synthase (SAMS) as these genes were induced in -S in both the mutant and the wild type. Genes from several categories including ‘response to hormone stimulus,’ ‘signal transduction,’ ‘defense response’ and ‘regulation of transcription’ genes were, however, misregulated in the mutant. Although many genes had different expression profiles in the KD several genes related to ethylene signaling (homologs of *Arabidopsis* EIN3-BINDING F BOX PROTEIN 1 (EBF1), ETHYLENE INSENSITIVE 4 (EIN4) and ETHYLENE RESPONSE SENSOR 1 (ERS1)) and ABA- and cytokine-mediated signaling (homologs of ARABIDOPSIS THALIANA HOMEOBOX 7 (ATHB-7) and HISTIDINE-CONTAINING PHOSPHOTRANSMITTER 1 (AHP1)) attracted particular attention (Moniuszko et al., 2013). The expression of these genes was slightly higher in the KD than in the wild type in sulfur-sufficient medium, but the most interesting effect was the very low expression of these genes in the KD line when plants were transferred to -S conditions. In *Arabidopsis* EBF1 is important for proteosomal degradation of ETHYLENE-INSENSITIVE3 (EIN3), the positive regulator of ethylene-responsive genes, whilst EIN4 and ERS1 are genes for ethylene receptors (Wang et al., 2006). These observations, along with the reduced ethylene level in the mutant grown in -S, prompted the authors to hypothesize that UP9C is involved in modulation of the ethylene signaling pathway, which is important in plant response to -S conditions. The main conclusion to be drawn from this work is that one of the functions of UP9C - and possibly also other LSU-like proteins - in plant response to -S may be related to the involvement of LSU-like proteins in tuning up ‘hormone stimulus’ signals induced by -S conditions. Although the authors focused on ethylene it is likely that other hormone signaling systems, possibly those involved in -S response, are also affected in the mutant.

## LSU-LIKE PROTEINS AND THEIR POTENTIAL INTERACTING PARTNERS

**FIGURE 3:**
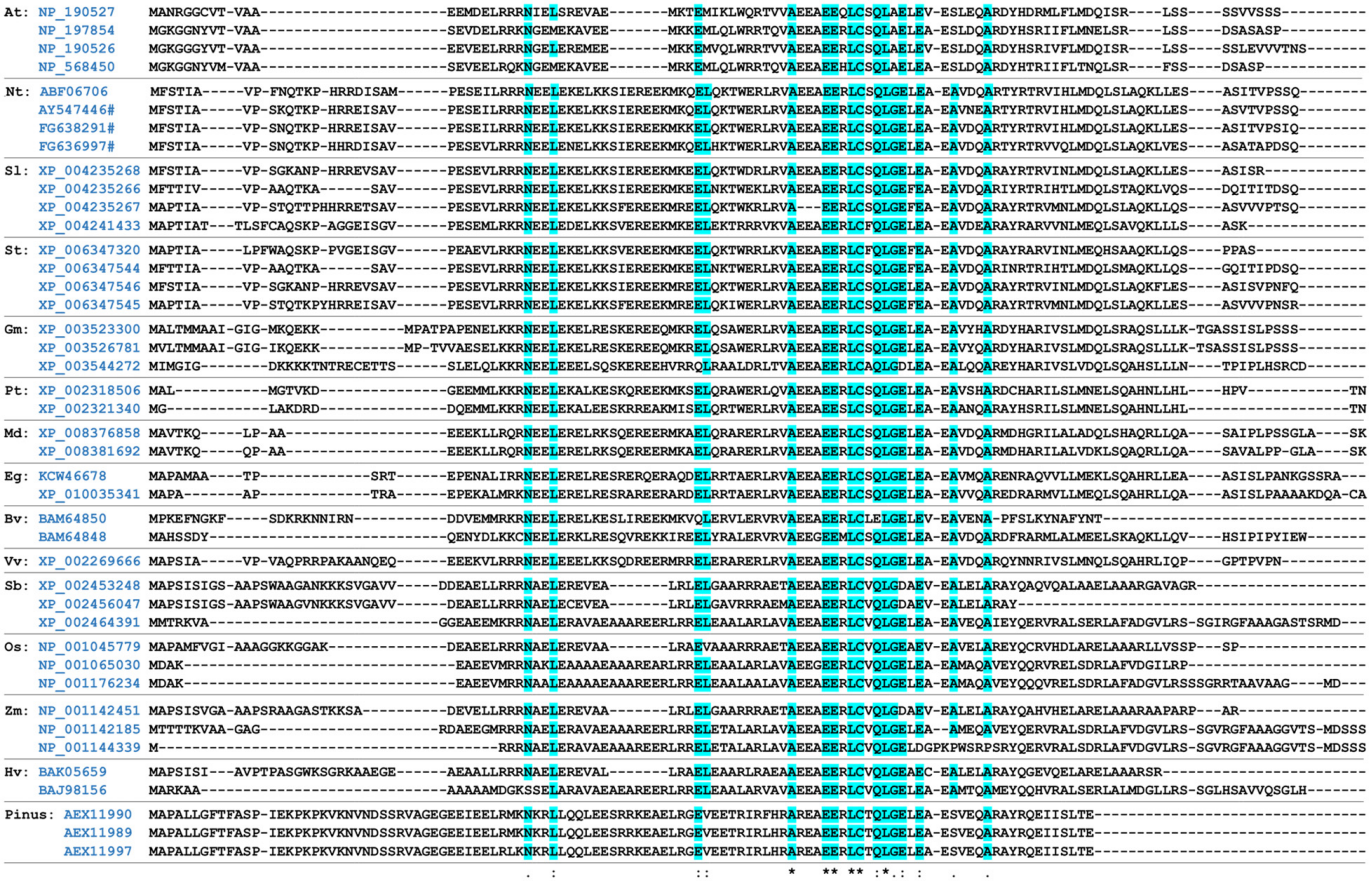
**Alignment of the selected LSU-like proteins.** The evolutionary conserved amino acids identified by the MAFT alignment software [http://mafft.cbrc.jp] are highlighted. The accession numbers of the protein sequences are provided. # denotes the accession number to the corresponding nucleotide sequence; At, *Arabidopsis thaliana*; Nt, *Nicotiana tabacum*; Sl, *Solanum lycopersicum*; St, *Solanum tuberosum*; Gm, *Glycine max*; Pt, *Populus trichocarpa*; Md, *Malus domesticus*; Eg, *Eucalyptus grandis*; Bv, *Beta vulgaris*; Vv, *Vitis vinifera*; Sb, *Sorghum bicolor*; Os, *Oryza sativa*; Zm, *Zea mays*; Hv, *Hordeum vulgare*; Pinus, *Pinus taeda.*

LSU/UP9 family proteins are small (10–13 kDa) and consist of about 100 amino acids (**Figure 3**). A BLAST (blastp) search of non-redundant protein sequences revealed multiple homologs of LSU in various plant species, both monocotyledons and dicotyledons, including *Solanum lycopersicum* (4 homologs), *Solanum tuberosum* (4), *Glycine max* (3), *Populus trichocarpa* (3), *Zea mays* (3), *Hordeum vulgare* (2), *Oryza sativa* (3), *Beta vulgaris* (2) and many others. The LSU-like proteins are also present in gymnosperms, like *Pinus* sp. We believe that so far only *Arabidopsis* LSUs and tobacco UP9s have been analyzed. Computer analysis and the circular dichroism spectra indicated that UP9C has an alpha-helical structure (Lewandowska et al., 2010). The presence of two stranded coiled–coil regions in UP9C (Lewandowska et al., 2010) is strongly suggestive of multimer formation; UP9C-UP9C interactions were observed in yeast two-hybrid (Y2H) experiments. Interestingly, despite relatively weak conservation of the primary sequence, both homologous UP9C-UP9C and heterologous LSU-UP9C (cross-species) interactions were observed. A potential nuclear localization signal was found in UP9C using the MOTIFSCAN program; according to PSORT UP9C has a cytosol-nuclear localization. No nuclear localization motifs have been identified in *Arabidopsis* LSU proteins. Nuclear localization of UP9C was reported (Lewandowska et al., 2010), but more recent experimental data suggest that it is present in both cytoplasm and nucleus (Moniuszko et al., 2013). Because they are small proteins it is likely that LSU-like proteins can cross the nuclear pores without a specific transport mechanism. There are no specific motifs or domains in LSU/UP9 proteins that suggest their function. The significance of the short, strongly evolutionarily conserved region in the members of this family (**Figure 3**) is unknown.

**FIGURE 4:**
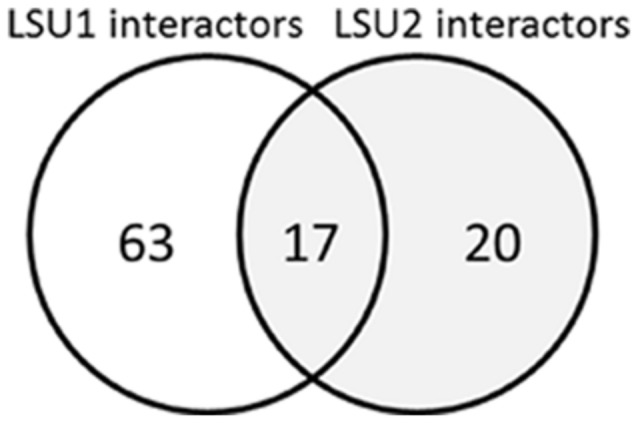
**Venn diagram of potential LSU1 and LSU2 interacting partners (*Arabidopsis* Interactome Mapping Consortium, 2011)**.

The LSU/UP9 proteins seem to be involved in multiple protein–protein contacts (**Table 1**; **Figure 4**). Data from tobacco are limited; however, some of the interacting partners identified by the Y2H approach have been confirmed using other methods. For example, UP9C interacts with ACO2A, an enzyme which converts 1-aminocyclopropane-1-carboxylate (ACC) to ethylene; it was therefore proposed that ethylene production might be controlled by UP9C through its interactions with ACO2A (Moniuszko et al., 2013). Joka2/NBR1 functions as a cargo receptor in selective autophagy (Zientara-Rytter et al., 2011) and is another protein which is unquestionably involved in interactions with UP9/LSU; however, the biological significance of these interactions is as yet unexplained.

**Table 1:** Tobacco proteins found as interacting with tobacco UP9C.

Accession number	Clone name	Number of amino acids in the clone	Identification/Function	Corresponding *A. thaliana* gene
**Library from *Nicotiana plumbaginifolia* seedlings grown in S-sufficient conditions**
ABF06703	NpJoka2	467	NBR1-like, cargo receptor of selective autophagy	At4g24690
ABF06705	NpJoka8	360	HLH superfamily; bHLH66	At2g24260
ABF06704	NpJoka20	161	Ribosomal L7/L12	At3g27850 At4g36420 At4g37660
**Library from 3-month-old *Nicotiana tabacum* plants transfered for 2 days into S-deficient conditions**
GU066878	Joka 31A	117	ACC oxidase	At1g05010
GU066879	Joka 31B	56	ACC oxidase	At1g05010
GU066880	Joka 32	253	PRP11; ZnF-U1 – splicing factor	At2g32600
GU066881	Joka 33	245	TIM50 (mt-inner membrane)	At1g55900
GU066882	Joka 34	376	RING-finger-containing E3 ubiquitin ligase	At3g58030
GU066883	Joka 35	147	RING-finger-containing E3 ubiquitin ligase	At3g16720
GU066884	Joka 36	314	Apetala 2-like (tran At4g36920 At5g67180 At5g60120
GU066885	Joka 37	110	Function unknown; Involucin repeat; phosphoenolopyruvate carboxylase; E2-enzyme	At2g28540 At3g55720
GU066886	Joka 38	144	DUF248/methyltransferase	At4g18030 At1g26850
GU066887	Joka 39	119	DUF632/Function unknown, leucine zipper	At2g27090
GU066888	Joka 40	515	Function unknown, nucleoporin-like	At4g37130
GU066889	Joka 41	99	Poly A binding	At1g49760 At4g34110 At2g23350 At1g22760 At1g71770
GU066890	Joka 42	77	FtsH protease	At2g26140
GU066891	Joka 43	128	Unknown	At3g24506 At2g17240
GU066892	Joka 44	75	Microtubule-associated MAP65-1a	At5g55230 At4g26760
GU066893	Joka 46	184	CHORD, PBS2, RAR1, interacts with SGT1; Rar1/TMV resistance	At5g51700
GU066894	Joka 47	200	JAZ1 (transcription factor)	At1g19180

**FIGURE 5:**
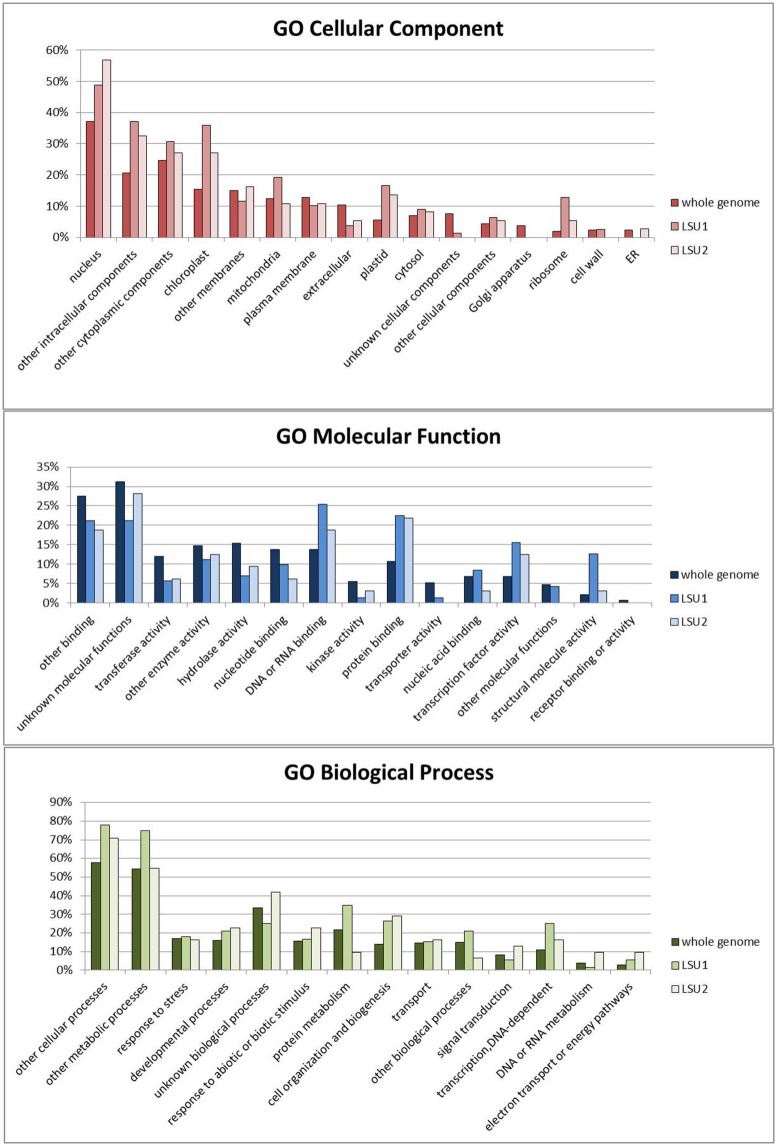
**Functional categorization of the potential LSU1 and LSU2 interacting partners for GO Cellular Component, GO Molecular Function and GO Biological Process.** The analysis was done using the Gene ontology tools available at TAIR [http://www.arabidopsis.org].

Mapping of the *Arabidopsis* interactome based on the Y2H system (Arabidopsis Interactome Mapping Consortium, 2011) has revealed numerous partners of LSU1 and LSU2; unfortunately LSU3 and LSU4 were not included in the experiments. The lists of proteins which potentially interact with LSU1 or LSU2 are quite long (80 and 37 proteins, respectively) and include 17 elements common to both proteins (**Figure 4**). Functional categorization of potential interacting partners using GO analysis indicated some changes in the distribution of gene product locations, molecular functions and biological processes relative to those for the genome as a whole (**Figure 5**). Both groups (LSU1 and LSU2 interacting partners) were more likely than average to be located in the nucleus, chloroplasts (plastids) or ribosomes. Nuclear proteins which are LSU1 or LSU2 interacting partners include members of the JAZ family of repressors. It is worth noting that it has been demonstrated that the tobacco homolog of JAZ interacts with UP9C (**Table 1**). Molecular Functions GO categories such as ‘DNA or RNA binding,’ ‘protein binding’ and ‘TF activity’ are over-represented among LSU1 and LSU2 interacting partners, whereas categories related to some enzymatic activities are under-represented.

We have also noticed that the group of LSU1 interacting partners includes a relatively high proportion of proteins from the Molecular Function GO category ‘structural molecule activity’ (all are ribosomal proteins). Overrepresentation of any Biological Process GO category was less apparent; ‘cell organization and biogenesis’ and ‘DNA-dependent transcription’ and perhaps the ‘protein metabolism’ and ‘transcription, DNA-dependent’ categories were only slightly overrepresented among LSU1 partners. The category of ‘cell organization and biogenesis’ proteins which interact with LSU1 includes some ribosomal proteins, chaperones and members of RING superfamily (potential E3 ubiquitin ligases). LSU1 partners include, amongst others, members of the ERF/AP2, bHLH and myb-like HTH families of transcriptional factors.

It has also been demonstrated that LSU2 protein interacts with the pathogenic effectors of two different plant pathogens, the bacterium *P. syringae* and the oocyte *Hyaloperonospora arabidopsidis* (Mukhtar et al., 2011). The involvement of LSU2 in the immune response to these pathogens was verified by the same authors through the demonstration of enhanced susceptibility in *lsu2* mutants (see also above).

## CONCLUDING REMARKS

It is unclear why plants have several isoforms of LSU. The proteins have probably partially overlapping functions; however, the data reported above suggest some functional specificity. *LSU1, LSU2,* and *LSU3* genes from *Arabidopsis* are induced by sulfur deficiency; however, only *LSU2* has been shown to be involved in retrograde signaling associated with chloroplast malfunction. The molecular role of LSU-like family members remains unclear although an increasing amount of evidence links the family with complex intracellular regulatory functions and coordination of organellar and cytosolic metabolism. It is possible that LSU/UP9 proteins modulate degradation of some specific “strategic” targets (such as TFs) in response to environmental stresses or are (directly or indirectly) involved in regulation of cellular degradation machinery. Although there is no clear evidence that LSU-like family members play such roles their interactions with presumed E3 ubiquitin ligases, chaperons (DnaJ-domain, Hsp60) and particularly with NBR1 (a selective autophagy cargo receptor) make the hypothesis plausible.

## AUTHOR CONTRIBUTIONS

Agnieszka Sirko and Anna Wawrzynska drafted the manuscript. Milagros Collados Rodríguez and Pawel, Skektas contributed to the writing process and preparation of figures. All authors were involved in preparing the final version.

## Conflict of Interest Statement

The Associate Editor Stanislav Kopriva declares that, despite having edited a Research Topic with the author Agnieszka Sirko, the review process was handled objectively and no conflict of interest exists. The authors declare that the research was conducted in the absence of any commercial or financial relationships that could be construed as a potential conflict of interest.
